# Carbon-Encased Mixed-Metal Selenide Rooted with Carbon Nanotubes for High-Performance Hybrid Supercapacitors

**DOI:** 10.3390/molecules27217507

**Published:** 2022-11-03

**Authors:** Yu Yuan, Panpan Cui, Jie Liu, Wei Ding, Yong Wang, Liping Lv

**Affiliations:** 1 School of Environmental and Chemical Engineering, Shanghai University, 99 Shangda Road, Shanghai 200444, China; 2 Key Laboratory of Organic Compound Pollution Control Engineering (MOE), Shanghai University, 99 Shangda Road, Shanghai 200444, China

**Keywords:** carbon nanotubes, supercapacitor, mixed-metal selenides, bimetallic organic framework

## Abstract

Transition metal-based compounds with high theoretical capacitance and low cost represent one class of promising electrode materials for high-performance supercapacitors. However, their low intrinsic electrical conductivity impedes their capacitive effect and further limits their practical application. Rational regulation of their composition and structure is, therefore, necessary to achieve a high electrode performance. Herein, a well-designed carbon-encased mixed-metal selenide rooted with carbon nanotubes (Ni-Co-Se@C-CNT) was derived from nickel–cobalt bimetallic organic frameworks. Due to the unique porous structure, the synergistic effect of bimetal selenides and the in situ growth of carbon nanotubes, the composite exhibits good electrical conductivity, high structural stability and abundant redox active sites. Benefitting from these merits, the Ni-Co-Se@C-CNT exhibited a high specific capacity of 554.1 C g^−1^ (1108.2 F g^−1^) at 1 A g^−1^ and a superior cycling performance, i.e., 96.4% of the initial capacity was retained after 5000 cycles at 10 A g^−1^. Furthermore, a hybrid supercapacitor assembled with Ni-Co-Se@C-CNT cathode and activated carbon (AC) anode shows a superior energy density of 38.2 Wh kg^−1^ at 1602.1 W kg^−1^.

## 1. Introduction

The growing consumption of traditional fossil fuels and the increasing requirement for the application of electric devices as well as large-scale energy storage stimulate researchers to pursue advanced and safe energy storage devices with both high energy and power density [1,2,3,4]. Among a variety of energy storage systems, supercapacitors (SCs) are representative due to high power density with the capability of fast charge and discharge process [5]. Generally, according to the difference in an energy storage mechanism, SCs can be mainly divided into electrochemical double-layer capacitors (EDLCs) and pseudocapacitors. For the EDLCs, storage of electrical energy is realized via electrostatic interactions at the electrode/electrolyte interfaces. Additionally, pseudocapacitors originate from faradic reactions on/near the surfaces of electrode materials. As the critical part of SCs, electrode materials play a decisive role in the energy storage process and the cyclability of devices [6].

Transition metal selenides (TMS) are widely used in the research of electrode materials for fuel cells, lithium-ion batteries and supercapacitors due to their redox activity [7]. Previous reports suggest that the electrochemical performance of single metallic selenide is not superior to bimetallic selenides due to the synergistic effects between bimetallic metal selenides, and the electronic structure and coupling interaction can be more probably adjusted [8,9,10,11,12,13]. Moreover, studies have shown that metallic selenides exhibit better electrochemical properties than their corresponding metallic oxides or sulfides due to their higher activities [13]. Therefore, bimetallic selenides are promising electrode materials for supercapacitors. Nevertheless, the low conductivity and limited exposure of active sites make their electrochemical activity not efficient to display. The increment of utilization on the redox sites of two metal selenides through rational design of the structure is therefore of great significance towards promoted electrochemical performances.

Metal–organic frameworks (MOFs) are porous crystalline materials with periodic network structures formed by metal ions and organic linkers through coordination bonds [13,14,15,16,17]. Various MOF derivatives can be produced by simple heat treatment with their inherent porous structure well-retained, and the decomposition of organic linkers further generates more porous structures. Moreover, metal chalcogenides can be easily obtained by the corresponding oxidation/vulcanization/selenylation [17,18,19]. Carbon layers generated from the pyrolysis of organic linkers can work as structural support and promote electron transportation for the electrode materials [20]. In order to strengthen the electrical conductivity of the MOF derivatives, the introduction of secondary carbon nanomaterials such as carbon nanotubes (CNTs) with excellent mechanical and electrical properties can be further suggested [21]. Moreover, originating from the structure of MOFs themselves, the metal centers such as Ni, Co and Fe are typical catalysts for in situ grow CNTs through chemical vapor deposition [22]. As compared to the simple compositing method to introduce CNTs, the in situ generation method can overcome the drawbacks such as uneven distribution and weak interaction between the active materials and carbon, playing better roles in promoting the performances of the electrodes.

Herein, by using a Ni-Co bimetallic organic framework precursor (Ni-Co-BTC, BTC = 1,3,5-homo-tricarboxylic acid), we synthesized a well-designed carbon-encased mixed-metal selenide rooted with carbon nanotubes (Ni-Co-Se@C-CNT) via thermal treatment process together with in situ generations of CNTs followed by selenylation. CNTs are in situ grown on the selenide spheres during carbonization due to the catalytic role of Ni and Co-based catalysts, which are generated upon the reduction atmosphere at high temperatures. In addition to the carbon layers arising from the organic linkers in MOFs, the CNTs rooted in Ni-Co-Se@C-CNT further accelerate the electron/ion transport and improve the structural stability. The porous structure and mixed metal selenides donate more exposed active sites and facilitate the interfacial contact between the electrode materials and the electrolytes. Consequently, the as-prepared Ni-Co-Se@C-CNT exhibits a high specific capacity and superior cyclability. Furthermore, a hybrid supercapacitor device assembled with Ni-Co-Se@C-CNT and activated carbon (AC) electrode shows high energy and power density.

## 2. Results and Discussion

**Figure 1:**
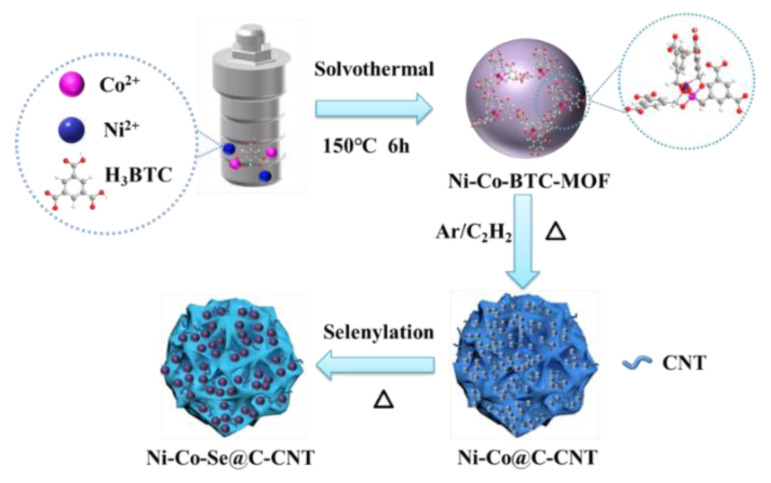
Schematic illustration of the growth process of Ni-Co-Se@C-CNT.

The preparation route of Ni-Co-Se@C-CNT is shown in Figure 1. The Ni-Co-BTC MOFs were obtained by coordinating nickel and cobalt ions with H_3_BTC using a hydrothermal method. Then, microsphere intermediates of carbon-supported Ni_3_C and Co_3_C rooted with carbon nanotubes were produced through an in situ chemical vapor process catalyzed by Ni and Co-based catalysts. Thereafter, the Ni-Co-Se@C-CNT microspheres were obtained by a further step of selenylation. Figure S1a–c show the SEM images of the Ni-BTC, Co-BTC and Ni-Co-BTC MOF precursors, which display uniform spherical morphology with diameters from submicron to micron. XRD patterns exhibited in Figure S1d indicate that all three Ni/Co-BTC-based MOFs possess amorphous structures since no obvious diffraction peaks were observed. The FTIR spectrum of the three MOFs (Figure S2) shows similar absorption peaks, which are mainly arising from the organic ligand BTC. Specifically, the absorption peaks around 1560 cm^−1^, 1437 cm^−1^ and 1368 cm^−1^ correspond to the stretching vibrations of C=C in the benzene rings of BTC. Moreover, the absorption band around 1619 cm^−1^ can be assigned to the C=O bonds in Ni-BTC, Co-BTC, or Ni-Co-BTC MOF. This absorption signal underwent a redshift compared to the initial C=O bonds in H_3_BTC, proving the coordination interaction between the Co/Ni metal centers and BTC in the three as-synthesized MOFs [23,24]. Ni@C-CNT and Co@C-CNT intermediates were obtained after thermal treatments of Ni-BTC and Co-BTC MOF at 500 °C for 30 min. The morphologies were characterized by SEM (Figure S3a,c), the sample still maintained the spherical morphology of the precursor of MOF after carbonization and CNTs were simultaneously generated during the carbonization due to the catalytic role of Co and Ni-based catalysts. XRD patterns (Figure S4) confirmed the presence of Ni_3_C and Co_3_C species, which are consistent with the diffraction peaks of Ni_3_C (PDF 06-0697) and Co_3_C (PDF 26-0450). In addition, Figure S3b and d indicate that the original microsphere morphology of the two samples was still maintained after selenylation.

According to previous similar reports relevant to CNT growth catalyzed by Ni or Co-based catalysts (Ni, Co, their alloys or compounds such as carbides) using C_2_H_2_ as carbon sources, the growth of CNT is assumed to experience three stages: induction (carburization), nucleation and growth following with the interaction between the Ni or Co-based catalysts and C_2_H_2_ [22,25]. Firstly, as indicated by the XRD patterns shown in Figure S4, metal carbides Ni_3_C and Co_3_C were generated in Ni-Co@C samples after pyrolysis of the Ni-Co MOF precursor. Nevertheless, Co and Ni nanoparticles may also be produced and then undergo carburization followed by the decomposition of the carbon source C_2_H_2_ gas [22]. After the formation of metal carbides, their surfaces prevent further carbon diffusion and decompose by segregating part of the carbon dissolved. The segregated carbon atoms form the first graphene layer, initiating the formation of carbon nanostructure (nucleation). Then, it creates a concentration gradient of carbon in the catalyst that allows more C_2_H_2_ to be decomposed. Finally, the released carbon atoms assemble into the graphene layers and form the body of CNT (growth) [22]. The yield of CNT can be controlled by adjusting the reaction time and flow rate of the carrier gas, i.e., Ar gas, in this work, which helps transport the carbon source of C_2_H_2_ because they affect the diffusion and deposition process of carbon atoms which are necessary to form CNT [26]. In the present work, a constant reaction time and flow rate of Ar/C_2_H_2_ gas were adopted, and we mainly investigated the performance difference of electrode materials with or without the generation of CNT and did not further control the yield of CNT and their influence on the electrode performances. As suggested by previous reports, an optimal content of CNT may exist since the too-low content of CNT cannot provide sufficient conductive support to the electrode, while a too-high content of CNT inevitably reduces the energy storage capacity per weight [27,28,29,30].

**Figure 2:**
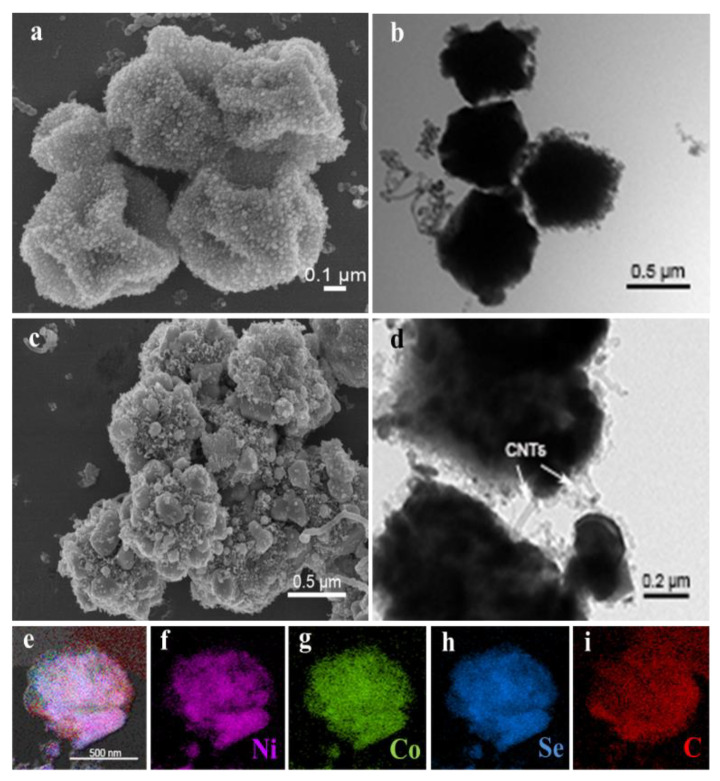
(**a**,**b**) SEM image and TEM image of Ni-Co@C-CNT; morphology characterization of Ni-Co-Se@C-CNT: (**c**) SEM image; (**d**) HRTEM image; (**e**–**i**) elemental mapping images.

The morphologies of Ni-Co@C and Ni-Co-Se@C are imaged in Figure S5. SEM (Figure S5a) and TEM (Figure S5b) images show that the diameter of Ni-Co@C microspheres was reduced to about 500 nm. This is mainly caused by thermal decomposition at high temperatures [31]. It is worth noting that SEM (Figure S5c) and TEM (Figure S5d) images of Ni-Co-Se@C show a wrinkled and rough microsphere morphology, which may be caused by the further step of selenylation. Note that due to the formation of selenides NiSe_2_ and CoSe_2_, the integrity of the Ni-Co-Se@C is destroyed to some extent as compared to Ni-Co@C. This morphology change inevitably affects the electron/ion transportation of the electrode and, consequently, the electrochemical performance during cycling. Nevertheless, Ni-Co-Se@C still kept its spherical morphology and did not collapse completely, and the in situ formed carbon materials arising from the MOF precursors can also work as the structural support for the system. The morphologies and nanostructure of the Ni-Co@C-CNT are shown in Figure 2a. Small nanoparticles and CNTs were grown on the irregular microspheres, which are due to the decomposition of C_2_H_2_ catalyzed by nano-nickel and nano-cobalt-based catalysts (Figure 2a). TEM image (Figure 2b) shows more clearly that CNTs were grown on the particles. According to the SEM image (Figure 2c), the structure of Ni-Co-Se@C-CNT microspheres changed after selenization. It can be observed that the surfaces roughened and became grainy, indicating a decrease in the microsphere integrity. Nevertheless, the microspheres still kept their spherical morphology, which can also be identified by the TEM image shown in Figure 2d. Additionally, according to EDS mapping data (Figure 2e–i), all the Ni, Co, Se and C elements in the Ni-Co-Se@C-CNT microsphere were observed to distribute uniformly. Due to the low contrast of CNT as compared to the metal selenides in SEM images and the small size of CNT (~20 nm), they are not obviously identified from the SEM images in Figure 2c. However, TEM images clearly show the presence of tubular CNT, as shown in Figure 2d. In comparison, the Ni-Co-Se@C without generation of CNT did not show any tubular CNT on the surfaces (Figure S5d).

**Figure 3:**
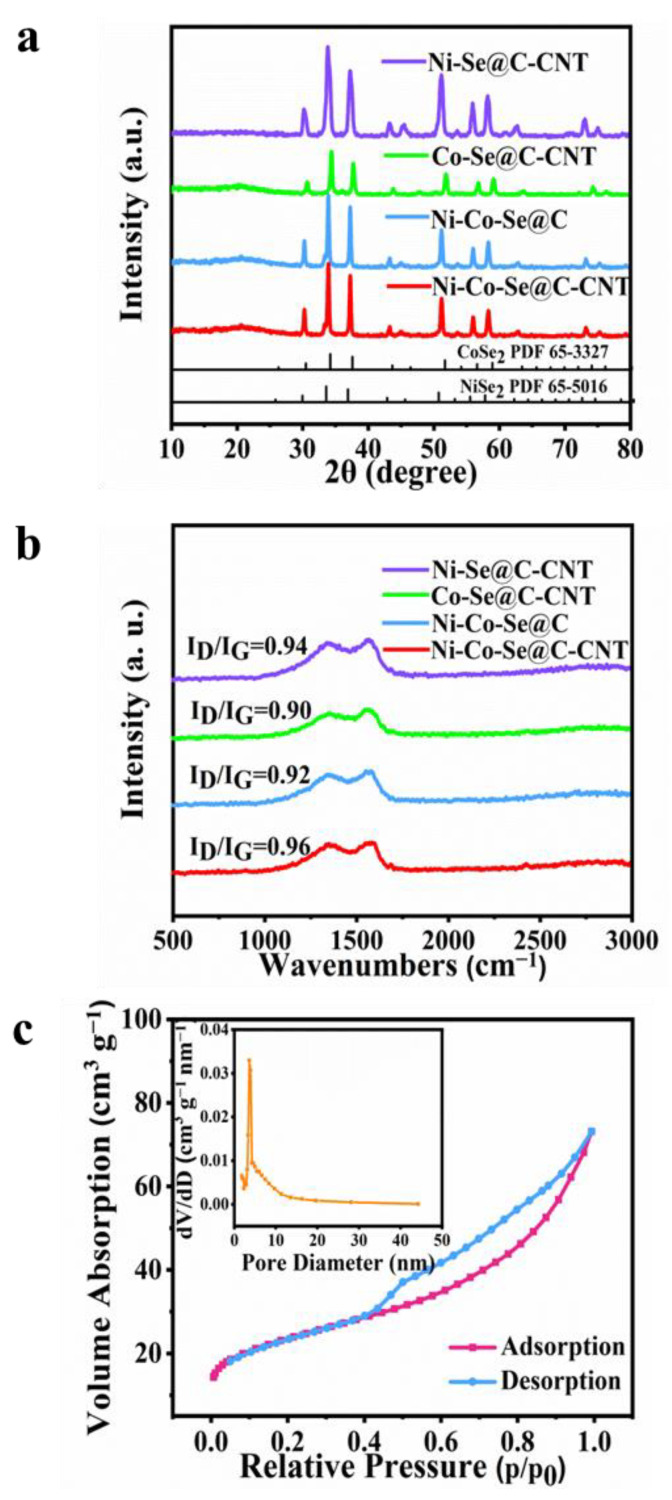
(**a**,**b**) XRD patterns and Raman spectra of Ni-Se@C-CNT, Co-Se@C-CNT, Ni-Co-Se@C and Ni-Co-Se@C-CNT. (**c**) Nitrogen adsorption-desorption isotherms and the inset showing the pore size distribution of Ni-Co-Se@C-CNT.

The crystallographic characteristics of the as-synthesized single or mixed metal selenides were further investigated by XRD (Figure 3a). The peaks at 30.2, 33.8, 37.2, 51.2, 55.9 and 58.2 correspond to NiSe_2_ phase (PDF 65-5016), while others diffraction signals at 30.6, 37.7, 51.9, 56.9 and 59.2 are indexed to CoSe_2_ (PDF 65-3327) [13]. Figure 3b further shows the Raman spectra of Ni-Se@C-CNT, Co-Se@C-CNT, Ni-Co-Se@C and Ni-Co-Se@C-CNT. The representative characteristic D and G bands of all the samples were observed at about 1327 cm^−1^ and 1580 cm^−1^, respectively [32,33]. Compared with the Ni-Co-Se@C-CNT (0.96), the I_D_/I_G_ values of Ni-Se@C-CNT (0.94), Ni-Co-Se@C (0.92) and Co-Se@C-CNT (0.90) were calculated to be lower, indicating more defects may exist in Ni-Co-Se@C-CNT, which would provide more active sites for its electrochemical storage. N_2_ adsorption–desorption isotherms (ADS) were evaluated to estimate the specific Brunauer–Emmett–Teller surface area (S_BET_), pore size and distribution of Ni-Co-Se@C-CNT (Figure 3c). The ADS profiles show a typical hysteresis loop of type IV in the scope of 0.4–1.0 P/P_0_ [34]. The S_BET_ of Ni-Co-Se@C-CNT was calculated to be about 81.3 m^2^ g^−1^. The pore size and distributions of Ni-Co-Se@C-CNT further confirm the presence of mesopores, and the majority of pore sizes fall in the range of 2 to 10 nm. The porous structure is beneficial to accelerate electron/ion diffusion dynamics and help improve its electrochemical energy storage characteristics. The contents of metal and selenium elements in Ni-Co-Se@C-CNT were tested by ICP-OES. The weight ratios of Ni, Co and Se in Ni-Co-Se@C-CNT were measured to be 14.98, 7.36 and 54.49 wt%, respectively. Therefore, the weight ratios of NiSe_2_ and CoSe_2_ were estimated to be 55.25 wt% and 27.05 wt%, respectively.

**Figure 4:**
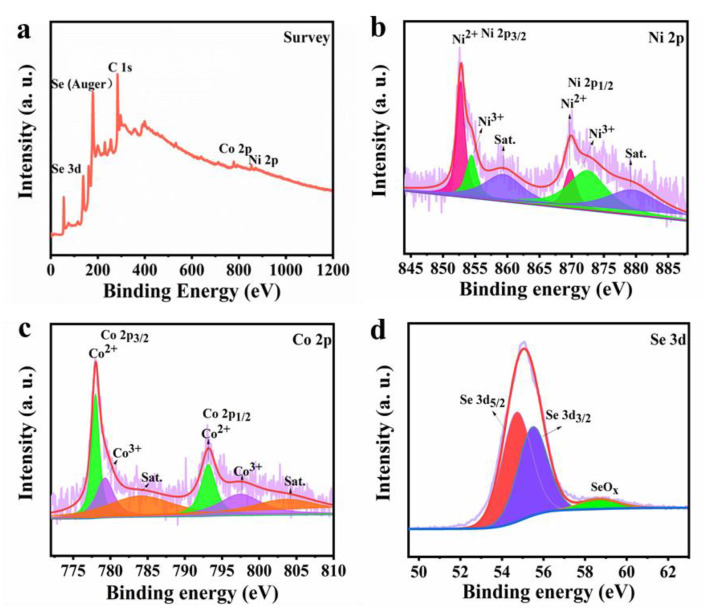
XPS spectra of Ni-Co-Se@C-CNT: (**a**) Survey; (**b**) Ni 2p; (**c**) Co 2p; (**d**) Se 3d.

The chemical states and composition of Ni-Co-Se@C-CNT were investigated by XPS technology. Figure 4a shows the overall survey scan of the Ni-Co-Se@C-CNT composite, indicating the presence of elements Co, Ni, Se and C. As for Ni 2p spectrum (Figure 4b), both Ni 2p_1/2_ and Ni 2p_3/2_ can be decomposed into three bands, the peaks at 869.8 (Ni 2p_1/2_) and 852.8 eV (Ni 2p_3/2_) can correspond to Ni^2+^. The peaks at 872.5 (Ni 2p_1/2_) and 854.4 eV (Ni 2p_3/2_) are attributed to Ni^3+^ in the surface oxide [35]. As for Co 2p spectrum (Figure 4c), the peaks at 793.1 (Co 2p_1/2_) and 778.1 eV (Co 2p_3/2_) can correspond to Co^2+^. The peaks at 779.2 eV (Co 2p_3/2_) and 797.4 eV (Co 2p_1/2_) are assigned to Co^3+^ [36]. Figure 4d further presents the Se 3d spectrum with peaks located at 54.8 eV and 55.5 eV for Se 3d_5/2_ and Se 3d_3/2_, respectively, while the main peak at 58.7 eV may be related to SeO_x_ [6]. This observation was consistent with the metal–selenium bonding in NiSe_2_ and CoSe_2_.

**Figure 5:**
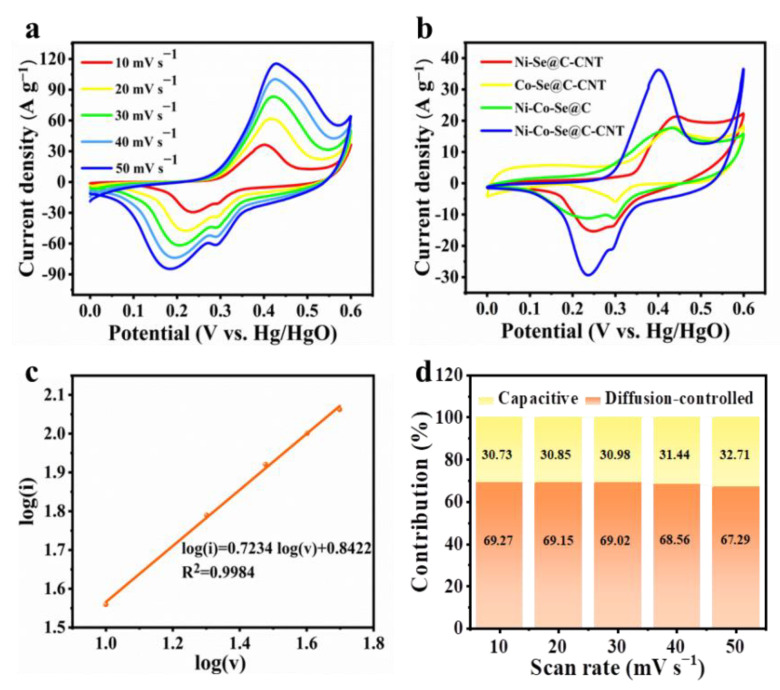
(**a**) CV curves of the Ni-Co-Se@C-CNT electrode at various scan rates; (**b**) CV curves of the Ni-Se@C-CNT, Co-Se@C-CNT, Ni-Co-Se@C and Ni-Co-Se@C-CNT electrodes at 10 mV s^−1^; (**c**) linear plot of log (i) versus log (v); (**d**) diffusion-controlled and capacitive contribution of Ni-Co-Se@C-CNT at various scan rates.

The electrochemical performance of the Ni-Co–Se@C-CNT was then evaluated in supercapacitors with 6 M KOH as the aqueous electrolyte (Figure 5). The distinct faradic reactions may correspond to the following Equations (1)–(6) [7,13,25,26]:

NiSe_2_ + H_2_O + 1/2O_2_ → Ni(OH)_2_ + 2Se (1)

CoSe_2_ + H_2_O + 1/2O_2_ → Co(OH)_2_ + 2Se (2)

3Se + 6OH^−^→ 2Se^2−^ + SeO_3_^2−^ + 3H_2_O (3)

Ni(OH)_2_ + OH^−^ ↔ NiOOH + H_2_O + e^−^ (4)

Co(OH)_2_ + OH^−^ ↔ CoOOH + H_2_O + e^−^ (5)

CoOOH + 2OH^−^ ↔ CoO_2_ + 2H_2_O + 2e^−^ (6)

NiSe_2_ and CoSe_2_ first convert to their corresponding hydroxides together with the generation of Se. The Se can react with OH^-^ to form Se^2−^ and SeO_3_^2−^. The metal hydroxides then convert to their hydroxide oxide in an alkaline solution with a redox conversion between Ni^2+^/Ni^3+^ and Co^2+^/Co^3+^. Then CoOOH can further convert to CoO_2_. The CV curves of Ni-Co-Se@C-CNT exhibit prominent redox peaks corresponding to the electrochemical transition of Ni^2+^/Ni^3+^ and Co^2+^/Co^3+^ with the assistance of OH^−^ (Figure 5a,b). With an increase in the scan rates, the redox peaks shift toward the positive and negative voltage, which may be related to the internal resistance of the electrode. Reaction kinetics and diffusion-controlled contribution of Ni-Co-Se@C-CNT were further evaluated. The analysis of the voltammetric response of the CV curves at varied scan rates can be described as follows [13]:

*i* = a*v*^b^ (7)

*i*(V) = k_1_*v* + k_2_*v*^1/2^ (8)

where *i* and *v*, respectively, are the current and scan rate, and a and b are adjustable parameters. k_1_ and k_2_ are constants. When b is close to 1, it indicates that the capacitive-controlled process mainly takes place at or near the surface of the electrode, while b approaches 0.5, which corresponds to a diffusion-controlled battery-type behavior. Based on the calculation, the value of b is 0.7234, implying a dominant diffusion-controlled process (Figure 5c). Figure 5b shows that the Ni-Co-Se@C-CNT possessed the largest CV integral area as compared to that of Ni-Se@C-CNT, Co-Se@C-CNT and Ni-Co-Se@C, indicating that it has a higher specific capacity.

In addition, the energy storage contributions arising from the capacitive and diffusion-controlled process can also be quantified using Equation (8). The capacitive contribution of the Ni-Co-Se@C-CNT electrode is estimated to be 30.73% at 10 mV s^−1^ (Figure 5d). As shown in Figure 5d, as the scan rates increase from 10 to 50 mV s^−1^, the capacitive-controlled process accounts from 30.73 to 32.71%. This observation reveals that the dominant charge-storing mechanism not only originated from the surficial part but also from the bulk materials, which are diffusion-controlled.

**Figure 6:**
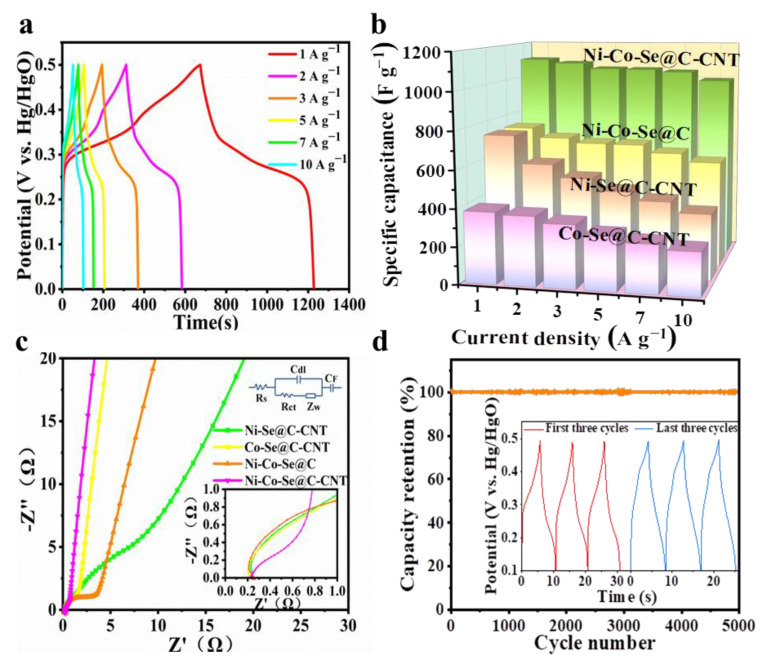
(**a**) GCD curves of the Ni-Co-Se@C-CNT electrode; (**b**) Specific capacities of Co-Se@C-CNT, Ni-Se@C-CNT, Ni-Co-Se@C and Ni-Co-Se@C-CNT electrode at different current densities; (**c**) EIS spectra of Ni-Se@C-CNT, Co-Se@C-CNT, Ni-Co-Se@C and Ni-Co-Se@C-CNT electrodes; (**d**) long-term cycling performance of Ni-Co-Se@C-CNT at 10 A g^−1^.

Figure 6a shows the galvanostatic charge–discharge (GCD) curves of Ni-Co-Se@C-CNT at various currents of 1–10 A g^−1^. The potential plateaus, which are consistent with the CV curves shown in Figure 5a, further indicate its battery-type behaviors. The specific capacities of Ni-Se@C-CNT, Co-Se@C-CNT, Ni-Co-Se@C and Ni-Co-Se@C-CNT were then calculated from GCD curves (Figure 6b and Figure S6). Ni-Se@C-CNT exhibited 368 C g^−1^ (736 F g^−1^) of capacity at 1 A g^−1^ but dramatically decayed at 10 A g^−1^, with 48.1% of capacity retention. Co-Se@C-CNT exhibited 190.6 C g^−1^ (381.2 F g^−1^) of capacity at 1 A g^−1^ but dramatically decayed at 10 A g^−1^, with 60.9% of capacity retention. Ni-Co-Se@C possessed 369.6 C g^−1^ (739.2 F g^−1^) of capacity at 1 A g^−1^ but slowly decayed at 10 A g^−1^, with only 77.7% of capacity retention. Remarkably, the Ni-Co-Se@C-CNT electrode exhibited a high specific capacity of 554.1 C g^−1^ (1108.2 F g^−1^) at 1 A g^−1^ with a superior 89.9% of capacity retention at 10 A g^−1^. Table S2 presents a comparative electrochemical performance of the Ni-Co-Se@C-CNT electrode with other previously reported bimetallic selenides. The superior specific capacity and better rate performance of the Ni-Co-Se@C-CNT electrode may be related to its small transfer resistance caused by the in situ growth of CNTs rooted in the system as well as the carbon matrix inherited from the bimetal–organic frameworks.

In order to better explore the reaction between electrode material and electrolyte at the interface, Electrochemical impedance spectroscopy (EIS) was tested. EIS plots and equivalent circuit models are shown in Figure 6c. Series resistance (Rs) was obtained from the intersection of the image and X axis; charge-transfer resistance (Rct) corresponds to the semicircle diameter in the high-frequency region; Warburg impendence (Zw) results from ions diffusion, which is related to the slope of the line during the low-frequency region. During the low-frequency region, the slope of the Ni-Co-Se@C-CNT was significantly larger than that of Ni-Se@C-CNT, Co-Se@C-CNT and Ni-Co-Se@C, indicating a lower diffusive resistance. By comparing with their semicircle diameter in the high-frequency region, the Rs and Rct values of different samples were estimated, i.e., Ni-Co-Se@C-CNT (0.244 Ω, 0.57 Ω), Ni-Se@C-CNT (0.248 Ω, 6.66 Ω), Co-Se@C-CNT (0.218 Ω, 1.88 Ω) and Ni-Co-Se@C (0.253 Ω, 2.44 Ω). All samples exhibit similar Rs values, while the Ni-Co-Se@C-CNT electrode (0.57 Ω) showed the lowest charge transfer resistance, which may be due to the higher electrical conductivity of the in situ growth of CNTs and the mixed metal selenide particles. Together with its higher I_D_/I_G_ value (Raman data), implying more defects to expose active sites and the mixed metal selenides that are assumed to provide abundant faradic sties compared to the single metal selenide, the Ni-Co-Se@C-CNT electrode finally displays the best electrochemical performance. The long-term cyclability of Ni-Co–Se@C-CNT was also investigated at 10 A g^−1^. Figure 6d and the inset figure indicate a capacity retention of about 96.4% after 5000 cycles, implying high structural stability of the Ni-Co–Se@C-CNT electrode.

**Figure 7:**
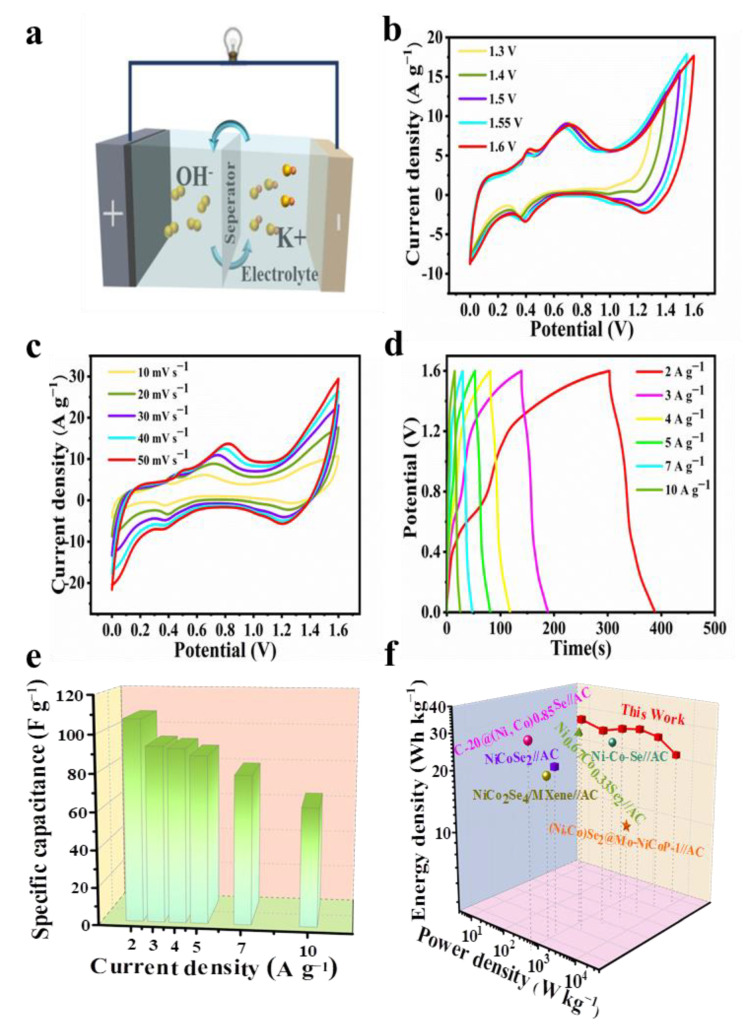
Electrochemical performance of the Ni-Co-Se@C-CNT//AC HSC: (**a**) schematic illustration of the assembled HSC; (**b**) CV curves with different cell voltages at 20 mV s^−1^; (**c**) CV curves of the HSC device at different scan rates; (**d**) GCD curves at different current densities; (**e**) the corresponding specific capacities of the HSC device; (**f**) Ragone plot of the as-assembled device and previously reported HSC.

Due to the superior electrochemical performance of the Ni-Co-Se@C-CNT in the three-electrode system, an HSC device with Ni-Co-Se@C-CNT and AC, respectively, as the cathode and anode, was assembled in 6 M KOH. Figure 7a is a schematic diagram of the assembled hybrid supercapacitor. The electrochemical performance of AC is shown in Figure S7. The AC electrode exhibited a typical electric double-layer capacitance (Figure S7a). Meanwhile, the specific capacity of AC was calculated to be 87.7 C g^−1^ (175.3 F g^−1^) at 1 A g^−1^ based on a three-electrode system (Figure S7b). Figure 7b shows the CV profiles of the HSC device operated with varied potential windows at 20 mV s^−1^. An observable oxygen evolution peak appeared when the potential working window was scaled up to 1.6 V. Therefore, a potential range between 0 and 1.6 V was applied for the device. Figure 7c shows the CV curve of Ni-Co-Se@C-CNT//AC HSC at 10–50 mV s^−1^. The curve shape remains basically unchanged even at different scanning rates. The results show that the device has good stability in the charging and discharging process. GCD curves of HSC at 2–10 A g^−1^ are shown in Figure 7d. Capacities of 53.7, 46.7, 46.2, 44.4, 39.7, 31.2 C g^−1^ (respectively, corresponding to 107.3, 93.4, 92.3, 88.8, 79.2 and 63.1 F g^−1^) were calculated from the GCD curves (Figure 7e) at 2, 3, 4, 5, 7 and 10 A g^−1^, respectively. Notably, more than 58.8% of the original capacity was maintained even at a high current density of 10 A g^−1^. Figure 7f shows the Ragone plot of the Ni-Co-Se@C-CNT//AC HSC device. Obviously, the fabricated Ni-Co-Se@C-CNT//AC HSC delivers a high energy density of 38.2 Wh kg^−1^ at a power density of 1602.1 W kg^−1^. The energy density still retains 22.4 W h kg^−1^ when the power density is up to 7987.3 W kg^−1^. The performance of our assembled device is superior to that of many recently reported devices such as C-20@(Ni, Co)_0.85_Se//AC [37], Ni_0.67_Co_0.33_Se_2_//AC [38], NiCoSe_2_//AC [39], Ni-Co-Se-2//AC [40], CuCo_2_Se_4_//AC [41] and NiCo_2_Se/MXene//AC [42]. These excellent electrochemical behaviors of the Ni-Co-Se@C-CNT electrode and its application in the HSC device can be summarized on account of the following factors. Firstly, the Ni-Co mixed-metal selenides can supply more faradic reactions compared to single-metal selenides. Secondly, the final three-dimensional structures basically maintain the spherical morphology of the precursors to prevent their agglomeration during long-term cycling. Lastly, the encased carbon arising from the organic ligands and in situ growth of CNT further provide a stable platform for the active sites as well as for the fast charge transfer and electron transport.

## 3. Experimental

### 3.1. Materials

Nickel nitrate hexahydrate (Ni(NO_3_)_2_ 6H_2_O, ≥98.0%, Sinopharm Chemical Reagent, Shanghai, China), Cobalt nitrate hexahydrate (Co(NO_3_)_2_ 6H_2_O, ≥98.5%, Sinopharm Chemical Reagent, Shanghai, China), N, N-dimethylformamide (DMF, 98%, Sinopharm Chemical Reagent, Shanghai, China), selenium (Se) powder (≥99.9%, Sinopharm Chemical Reagent, Shanghai, China) and 1,3,5-homotricarboxylic acid (H_3_BTC, 98%, Aladdin, Shanghai, China) were used as received without further purification.

### 3.2. Preparation of Ni-Co-BTC MOFs

A total amount of 1.75 mmol (0.5085 g) of Ni(NO_3_)_2_ 6H_2_O and 0.85 mmol (0.247 g) of Co(NO_3_)_2_ 6H_2_O were first dissolved in 30 mL of DMF. Then 1.4 mmol (0.294 g) of H_3_BTC and 1 g of polyvinylpyrrolidone (PVP) were added to the above mixture and stirred at room temperature for 1 h. The mixed solution was then transferred into a 50 mL Teflon-lined stainless autoclave maintained at 150 °C for 6 h. After natural cooling to room temperature, the obtained solids were washed with ethanol three times and dried overnight in an oven at 60 °C to obtain the final product Ni-Co-BTC MOFs. For comparison, Ni-BTC MOFs and Co-BTC MOFs were synthesized using 2.6 mmol (0.756 g) of Ni(NO_3_)_2_ 6H_2_O or Co(NO_3_)_2_ 6H_2_O and following the same synthesis procedure.

### 3.3. Preparation of Ni-Co@C-CNT Structures by Chemical Vapor Deposition

The as-synthesized Ni-Co-BTC MOFs were transferred to a tube furnace and annealed for 30 min at 500 °C with a heating rate of 3 °C min^−1^ in a mixed atmosphere of 5% C_2_H_2_ and 95% Ar. After natural cooling to room temperature, the intermediates of Ni-Co@C-CNT were collected. Ni@C-CNT and Co@C-CNT were obtained following the same method as control samples. Additionally, Ni-Co-BTC MOFs were calcined in Ar gas instead of C_2_H_2_/Ar to obtain the Ni-Co@C intermediate without the generation of CNTs.

### 3.4. Preparation of Ni-Co-Se@C-CNT

The Ni-Co@C-CNT and Se powder (1:3, *w*/*w*) were loaded into a combustion vessel separately, with the Se powder placed at the flow upstream within a tube furnace for 2 h under N_2_ gas flow at 500 °C (heating rate: 3 °C min^−1^). After natural cooling, the black product was collected and donated as Ni-Co-Se@C-CNT. Ni-Co-Se@C, Ni-Se@C-CNT, and Co-Se@C-CNT were also prepared in the same way.

### 3.5. Materials Characterization

The phase, composition and morphology information of the synthesized products were characterized with X-ray diffraction (XRD, Rigaku, D/MAX-2550V, Cu Kα rays, Japan, Tokyo). scanning/transmission electron microscopy (SEM: JEOL, JSM-6700F, Japan, Tokyo; TEM: JEOL, JEM-200CX, Japan, Tokyo). Raman spectroscopy (Renishaw, inVia pius, London, UK) and X-ray photoelectron spectroscopy (XPS, PerkinElmer, PHI ESCA-5000C, Waltham, MA, USA). The specific surface area and pore structure were evaluated on a surface area and porosimetry analyzer (Micromeritics Instrument Corp, ASAP 2020 M+C, Norcross, GA, USA). The contents of Ni, Co and Se elements were estimated using an inductively coupled plasma-optical emission spectrometer (PerkinElmer, Optima 2100DV, Waltham, MA, USA).

### 3.6. Electrochemical Measurements

The electrochemical performances of the as-synthesized samples were tested in a conventional three-electrode system (counter electrode: Pt; reference electrode: Hg/HgO; electrolyte: 6 M KOH) on a CHI760E electrochemical workstation. The voltage window for the CV and GCD test were set between 0~0.6 V and 0~0.5 V, respectively. Electrochemical impedance spectroscopy (EIS) was conducted from 0.01 Hz to 1000 kHz. A hybrid supercapacitor (HSC) device was fabricated using Ni-Co-Se@C-CNT as the cathode, activated carbon (AC) as anode and NKK TF40 (Nippon Kodoshi Corporation) as the separator. The charge balance was calculated based on the following equation [13]:

m_+_/m− = C− × ΔV−/(C_+_ × ΔV_+_) (9)

where m, C and ΔV, respectively, represents the mass, specific capacitance and the potential window of the cathode (+) or anode (−).

The specific capacitance of the electrode (C g^−1^ or F g^−1^) was calculated according to the equation shown as follows:

C_Q_ = I × Δt/m or C_F_ = I × Δt/(m × ΔV) (10)

where I, Δt, m and ΔV refer to the discharge current, time, weight of active materials and the potential window, respectively. The corresponding energy density (W h kg^−1^) and power density (W kg^−1)^ of the device can be estimated from the following equations:

E = 0.5C_F_ × ΔV^2^ (11)

P = 3600 × E/Δt(12)

## 4. Conclusions

In summary, a bi-MOF-derived Ni-Co-Se@C-CNT electrode was constructed by a facile method. The mixed metal selenides supply more active sites for faradic reactions compared to single-metal selenides. The in situ growth of CNTs further increases the electrical conductivity, accelerates electron and ion transport and further improves the structural stability of the electrode material. The Ni-Co-Se@C-CNT shows a superior cycling performance, retaining 96.4% of initial capacity after 5000 cycles at 10 A g^−1^. When applied as a cathode in supercapacitors, the assembled Ni-Co-Se@C-CNT//AC HSC delivers a superior energy and power density of 38.2 Wh kg^−1^ and 1602.1 W kg^−1^, respectively. The superior electrode performance of Ni-Co-Se@C-CNT can be attributed to the synergic effect of the mixed metal selenides that provide abundant faradic reactions and the 3D carbon support that arises from the organic ligands of the bimetallic organic framework to offer a conductive matrix towards fast electron/ion transportation. Additionally, the in situ generations of the CNTs rooted in the Ni-Co-Se@C spheres further provide a stable and conductive platform for the active sites toward the high-energy storage process.

## Data Availability

The data presented in this study are available on request from the corresponding authors.

## Supplementary Materials

The following supporting information can be downloaded at: https://www.mdpi.com/article/10.3390/molecules27217507/s1. Figure S1: SEM images of (a) Ni-BTC MOF, (b) Co-BTC MOF, and (c) Ni-Co-BTC MOF; (d) XRD patterns of Ni-BTC MOF, Co-BTC MOF, and Ni-Co-BTC MOF; Figure S2: FTIR spectra of Ni-BTC MOF, Co-BTC MOF, and Ni-Co-BTC MOF; Figure S3: SEM images of (a) Ni@C-CNT, (b) Ni-Se@C-CNT, (c) Co@C-CNT, and (d)Co-Se@C-CNT; Figure S4: XRD patterns of Ni@C-CNT, Co@C-CNT, Ni-Co@C, and Ni-Co@C-CNT; Figure S5: SEM images of (a) Ni-Co@C, (c) Ni-Co-Se@C; TEM images of (b) Ni-Co@C, (d)Ni-Co-Se@C; Figure S6: CV curves of (a) Ni-Se@C-CNT, (c) Co-Se@C-CNT, (e) Ni-Co-Se@C electrodes at various scan rates; GCD curves of (b) Ni-Se@C-CNT, (d) Co-Se@C-CNT, (f) Ni-Co-Se@C electrodes; Figure S7: Electrochemical properties of AC: CV curves; (b) GCD curves; Table S1: Results of the elemental content measurements of Ni-Co-Se@C-CNT; Table S2 [43,44,45,46,47,48,49,50,51]: Electrochemical performance comparison of bimetallic metal-selenide based supercapacitor electrodes. (SC: specific capacitance; RP: rate performance; CP: cycling performance.).

## Sample Availability

Samples produced in this work are available from authors.
