# Supplementary material for: Carbon-Encased Mixed-Metal Selenide Rooted with Carbon Nanotubes for High-Performance Hybrid Supercapacitors
Source: Molecules. 2022 Nov 3;27(21):7507. doi: 10.3390/molecules27217507 (PMC9656863; doi:10.3390/molecules27217507)
Supplement: Supplementary file 1 [file molecules-27-07507-s001.zip › molecules-1942069-supplementary.pdf]

Supporting Information for

Carbon-Encased Mixed-metal Selenide Rooted with Carbon Nanotubes for High-Performance Hybrid Supercapacitors

Yu Yuan[1], Panpan Cui[1], Jie Liu[1], Wei Ding[1], Yong Wang[*1,2], Li-Ping Lv[*1,2]

[1]*School of Environmental and Chemical Engineering, Shanghai University, 99 Shangda Road, Shanghai, P. R. China, 200444*

[2]*Key Laboratory of Organic Compound Pollution Control Engineering (MOE), Shanghai University, 99 Shangda Road, Shanghai, P. R. China, 200444*

*Corresponding authors: Tel: +86-21-66137723; fax: +86-21-66137725.

Email address: yongwang@shu.edu.cn; liping_lv@shu.edu.cn

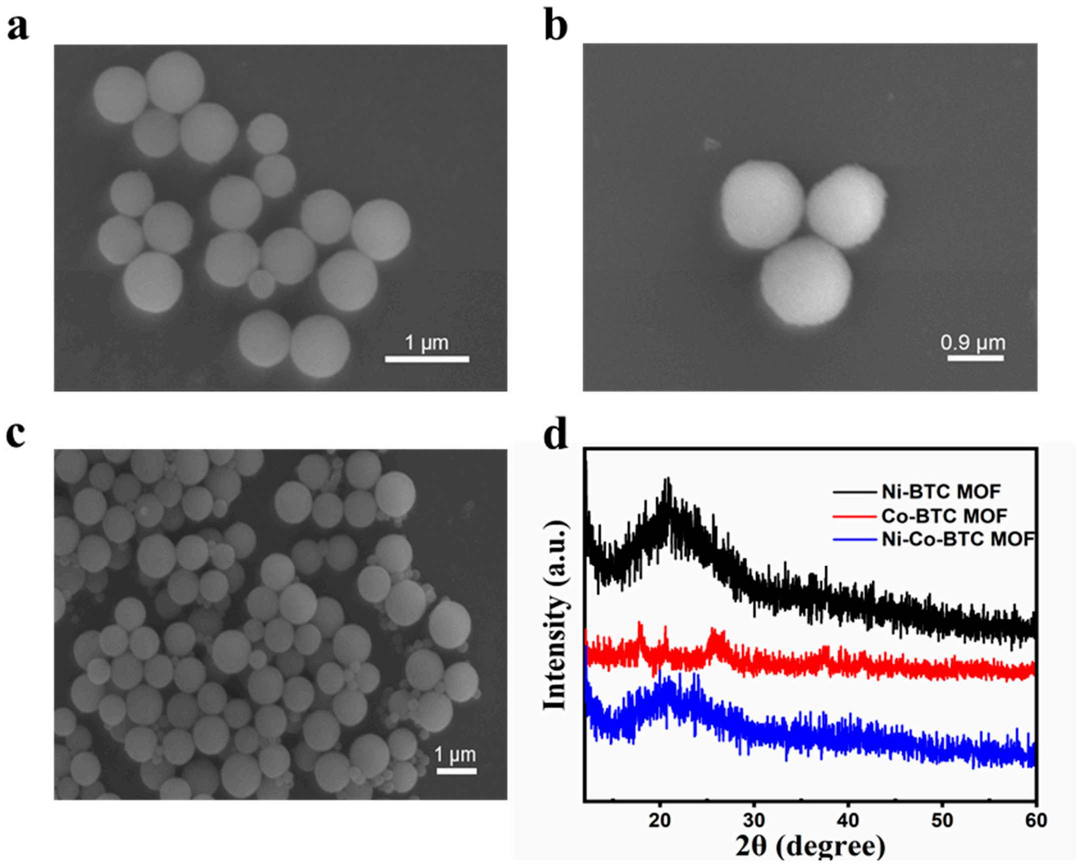

Figure S1. SEM images of (a) Ni-BTC MOF, (b) Co-BTC MOF, and (c) Ni-Co-BTC MOF; (d) XRD patterns of Ni-BTC MOF, Co-BTC MOF, and Ni-Co-BTC MOF.

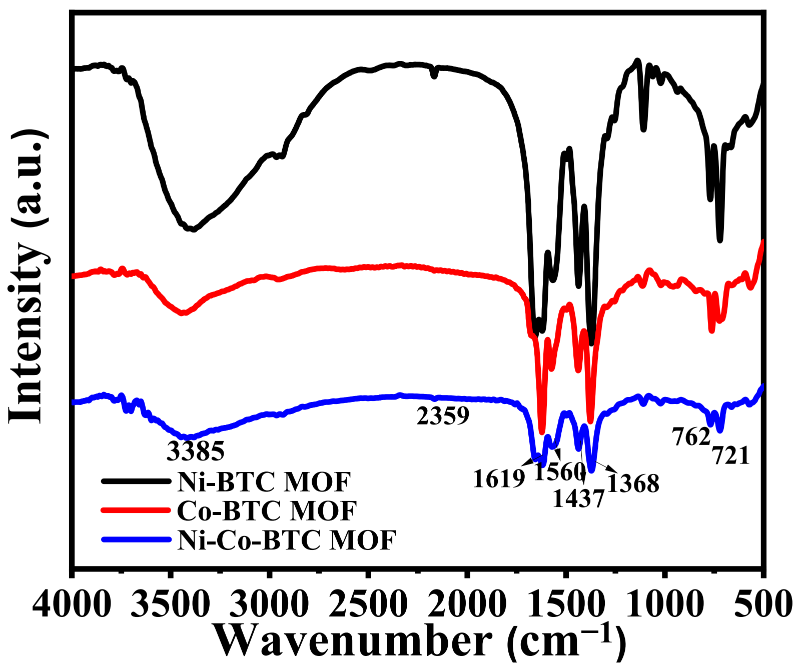

Figure S2. FTIR spectra of Ni-BTC MOF, Co-BTC MOF, and Ni-Co-BTC MOF.

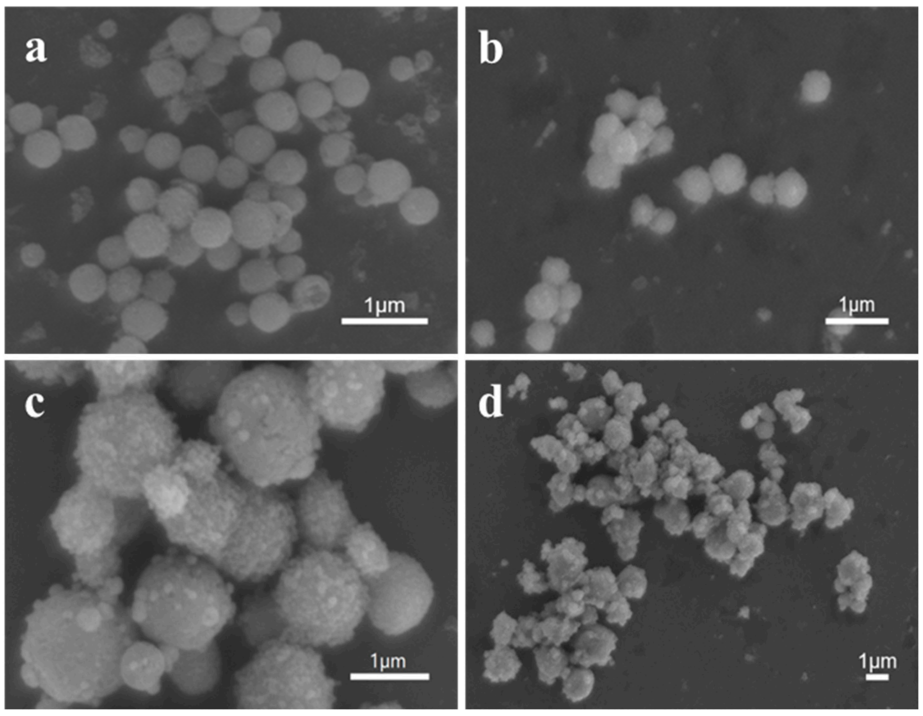

Figure S3. SEM images of (a) Ni@C-CNT, (b) Ni-Se@C-CNT, (c) Co@C-CNT, and (d) Co-Se@C-CNT.

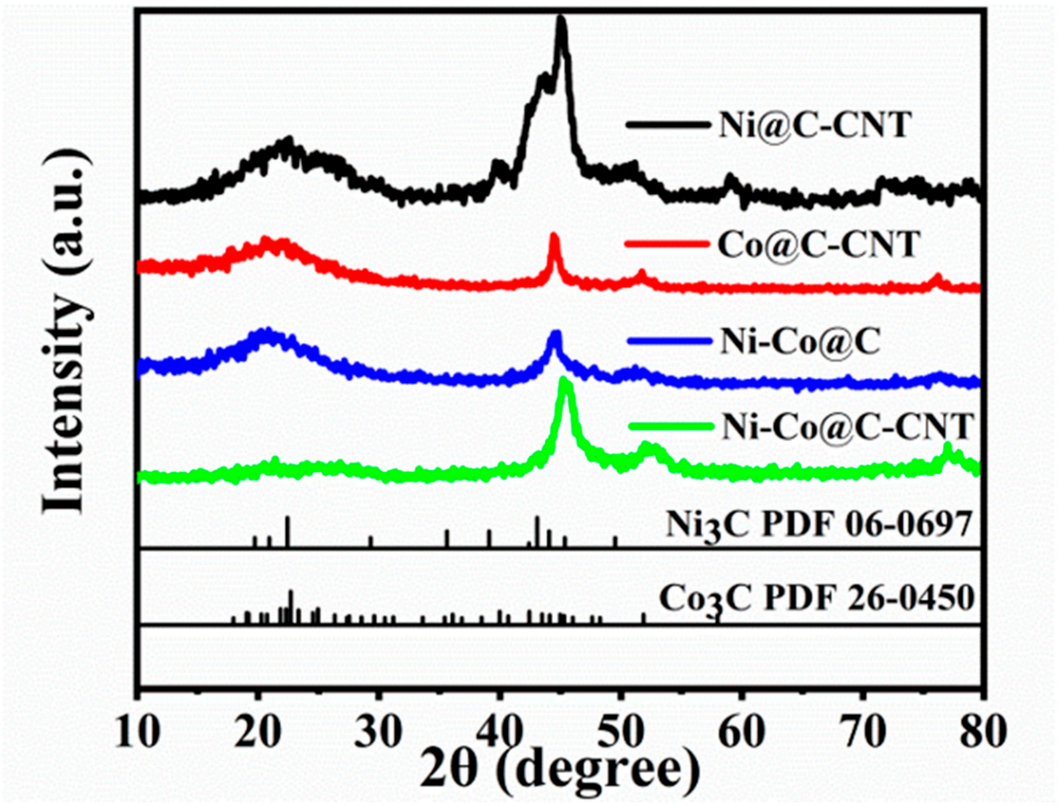

Figure S4. XRD patterns of Ni@C-CNT, Co@C-CNT, Ni-Co@C, and Ni-Co@C-CNT.

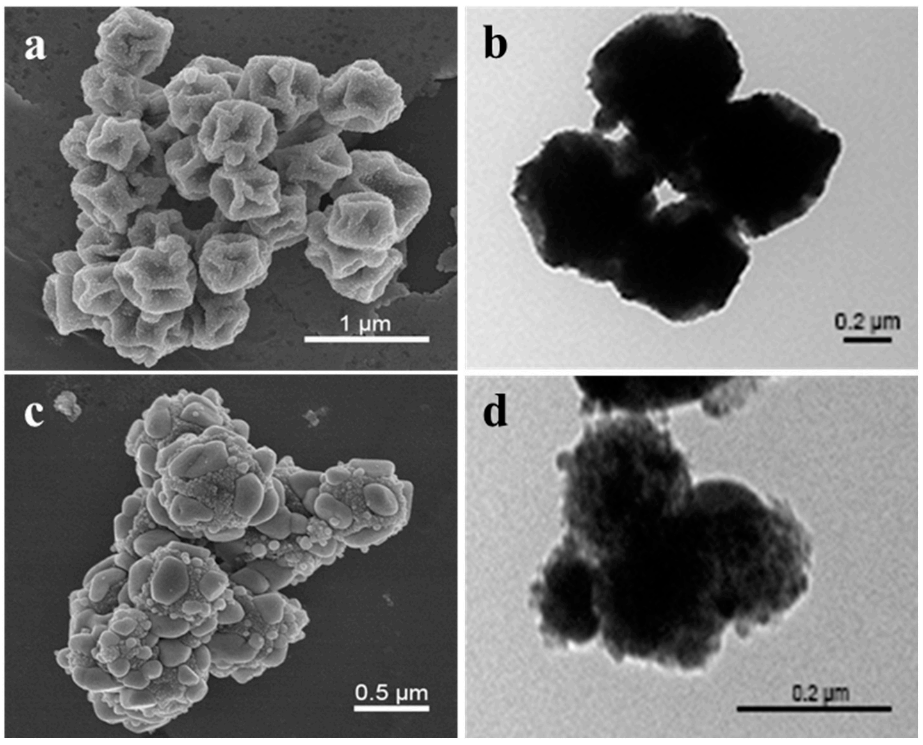

Figure S5. SEM images of (a) Ni-Co@C, (c) Ni-Co-Se@C; TEM images of (b) Ni-Co@C, (d) Ni-Co-Se@C.

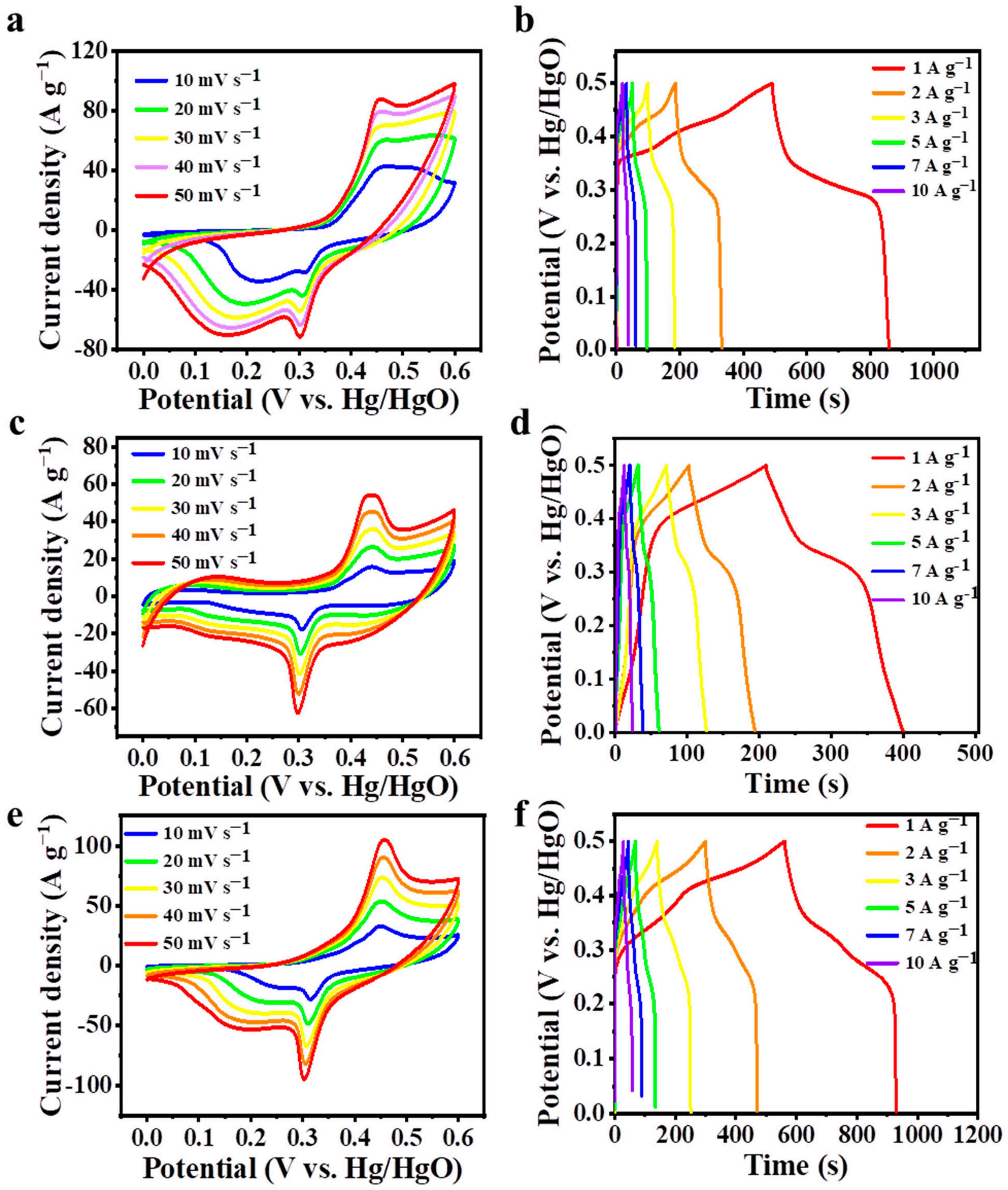

Figure S6. CV curves of (a) Ni-Se@C-CNT, (c) Co-Se@C-CNT, (e) Ni-Co-Se@C electrodes at various scan rates; GCD curves of (b) Ni-Se@C-CNT, (d) Co-Se@C-CNT, (f) Ni-Co-Se@C electrodes.

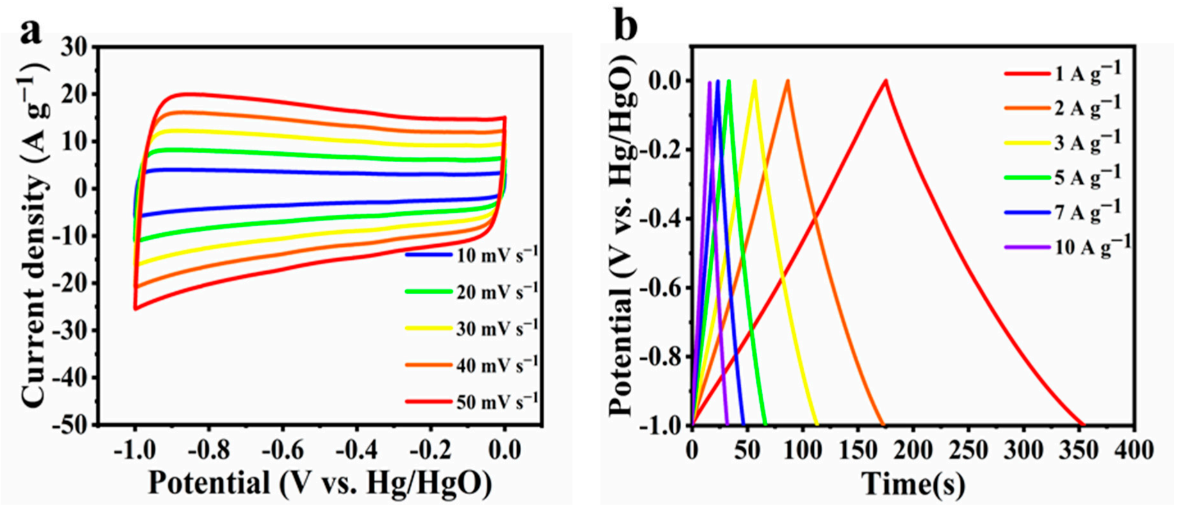

Figure S7. Electrochemical properties of AC: CV curves; (b) GCD curves.

Table. S1 Results of the elemental content measurements of Ni-Co-Se@C-CNT

Element	Mass content (%)
**Ni**	**14.98**
**Co**	**7.36**
**Se**	**54.49**

Table S2. Electrochemical performance comparison of bimetallic metal-selenide based supercapacitor electrodes. (SC: specific capacitance; RP: rate performance; CP: cycling performance.)

Ref.	Composite	SC	RP	CP
This work	Ni-Co-Se@C-CNT	554.1 C g^{-1} (1108.2 F g^{-1}) (1 A g^{-1})	89.9% (10 A g^{-1})	96.4 % (5000 cycles)
1	$(Ni,Co)Se_2$@rGO[43]	649.1C g^{-1} （1 A g^{-1}）	75.5% (20 A g^{-1})	90.5 % (5000 cycles)
2	$(Ni,Co)Se_2$-T[44]	636.2C g^{-1} （1 A g^{-1}）	66.1% (20 A g^{-1})	/
3	$(Ni,Co)Se_2$@MoNiCoP[45]	797C g^{-1} (2mA cm^{-2})	77.3% (50mA cm^{-2})	79.2 % (3000 cycles)
4	$CuCo_2Se_4$ Microspheres[41]	512 F g^{-1} (1 A g^{-1})	70.8% (24 A g^{-1})	83.7 % (6000 cycles)
5	((CuCo)Se/NC)[49]	121.4 C g^{-1} (1 A g^{-1})	82% (10 A g^{-1})	129.9 % (1200 cycles)
6	CF@(Ni,Co)Se-2[46]	188.8 mAh g^{-1} (1 A g^{-1})	58 % (20 A g^{-1})	/
7	$NiCo_2Se_4$/Mxene[42]	953.8 F g^{-1} (1 A g^{-1})	86.1% (9 A g^{-1})	93.9% (3000 cycles)
8	Ni-Co-Se-2[40]	461 F g^{-1} (1 A g^{-1})	76% (20 A g^{-1})	86.5 % (2000 cycles)
9	$NiCoSe_2$ spheres[39]	685 F·g^{-1} (1 A g^{-1})	50.9% (30A g^{-1})	/
10	$(Ni,Co)Se_2$ microspheres[12]	106 mAh g^{-1} (1 A g^{-1})	75% (10 A g^{-1})	78 % (5000 cycles)
11	$NiCu(OH)_2$@Ni-Cu-Se[47]	264.91 F g^{-1}(1 A g^{-1})	41.3% (10 A g^{-1})	75.8% (3000 cycles)
12	Ni-Co-Se nano-polyhedrons[6]	1668 F g^{-1} (1 A g^{-1})	82.8% (20 A g^{-1})	87.2 % (5000 cycles)
13	Zn–Co–Se/NF[51]	313.45 C g^{-1} (0.5 A g^{-1})	95% (10 A g^{-1})	93% (3000 cycles)
14	$(Ni,Co)Se_2/Ni(OH)_2$[48]	178.8 mAh g^{-1} (3 mA cm^{-2})	76.4% (30A g^{-1})	84.6% (3000 cycles)
15	Co–Mo–Se nanosheet arrays [50]	221.7 mAh g^{-1} (1 A g^{-1})	72% (20 A g^{-1})	95% (8000 cycles)
